# Geochemical Fractionation and Source Identification of Pb and Cd in Riparian Soils and River Sediments from Three Lower Reaches Located in the Pearl River Delta

**DOI:** 10.3390/ijerph192113819

**Published:** 2022-10-24

**Authors:** Shaowen Xie, Chengshuai Liu, Bin He, Manjia Chen, Ting Gao, Xinghu Wei, Yuhui Liu, Yafei Xia, Qianying Sun

**Affiliations:** 1 School of Environmental and Chemical Engineering, Foshan University, Foshan 528000, China; 2 Guangdong Laboratory for Lingnan Modern Agriculture, Guangzhou 510642, China; 3 National-Regional Joint Engineering Research Center for Soil Pollution Control and Remediation in South China, Guangdong Key Laboratory of Integrated Agro-Environmental Pollution Control and Management, Institute of Eco-Environmental and Soil Sciences, Guangdong Academy of Sciences, Guangzhou 510650, China; 4 State Key Laboratory of Environmental Geochemistry, Institute of Geochemistry, Chinese Academy of Sciences, Guiyang 550081, China; 5 South China Institute of Environmental Sciences, Ministry of Ecology and Environment, Guangzhou 510655, China

**Keywords:** riparian soils, river sediments, Pb and Cd, source identification, Guangdong–Hong Kong–Macao Greater Bay Area

## Abstract

Pb and Cd accumulation in riparian soils and river sediments in river basins is a challenging pollution issue due to the persistence and bioaccumulation of these two trace metals. Understanding the migration characteristics and input sources of these metals is the key to preventing metal pollution. This study was conducted to explore the contents, geochemical fractionation, and input sources of Pb and Cd in riparian soils and river sediments from three lower reaches of the Pearl River Delta located in the Guangdong–Hong Kong–Macao Greater Bay Area. The total concentration of all Pb and Cd values exceeded the background values to varying degrees, and the exchangeable fraction of Cd in riparian soils and river sediments accounted for the largest proportion, while that of Pb was dominated by the residual fraction. Geoaccumulation index calculations showed that in the riparian soils, the average accumulation degree of Pb (0.52) in the Beijiang River (BJR) was the highest, while that of Cd (2.04) in the Xijiang River (XJR) was the highest. Unlike that in riparian soils, the maximum accumulation of Pb (0.76) and Cd (3.01) in river sediments both occurred in the BJR. Furthermore, the enrichment factor results also showed that Pb and Cd in the riparian soils and river sediments along the BJR were higher than those in the XJR and Dongjiang River (DJR). The relationship between enrichment factors and nonresidual fractions further proved that the enrichment factors of Cd were significantly correlated with the nonresidual fractions of Cd, which may imply various anthropogenic sources of Cd in the three reaches. Moreover, source identification based on principal component analysis (PCA) and Pb isotope ratio analysis indicated that riparian soils and river sediments have inconsistent pollution source structures. The PCA results showed that Pb and Cd were homologous inputs in the DJR, and there were significant differences only in the riparian soils and river sediments. Pb isotope tracing results further showed that the bedrock of high geological background from upstream may be the main reason for Cd accumulation in the XJR. However, the ultrahigh accumulation of Cd in the BJR is mainly caused by the input of the upstream mining and metallurgy industry. The control of upstream input sources will be the key to the prevention of trace metal pollution in these regions.

## 1. Introduction

Excessive accumulation of trace metals in riparian soils and river sediments is a challenging environmental issue due to their stability, persistence, and bioaccumulation in many river basins around the world [1,2]. In particular, the Pearl River Basin, which is located in the Guangdong–Hong Kong–Macao Greater Bay Area (GBA) of southern China, has relatively high concentrations of trace metals in soils and sediments compared to other areas in China [3,4]. A comprehensive regional assessment of spatiotemporal changes in soil pollution in the GBA from 1989–2018 showed that all the examined trace metals (As, Cd, Cr, Ni, Cu, Zn, Pb, and Hg) were slightly elevated compared to their background values, except Cd, Hg, and Cu, which showed moderate contamination [5]. The types and level of heavy metal pollutions in sediments and water of the GBA are changing due to the rapid economic development [6,7,8], mining and smelting activities [9], and the human activities [10]. As one of the most economically vibrant areas and an important region for waterborne commerce in China, the environmental security of the river basin is of great importance to the drinking water safety of the residents along the river and the economic construction of the GBA [11].

Riparian soils and river sediments are two significant components of river basin ecosystems. Riparian soils are the most important sinks of trace metals around riparian zones, which have aquatic–terrestrial ecotones with unique biological, biophysical, and landscape features [12]. River sediments usually act as major sinks for trace metals and account for 30–98% of the total metal loads in rivers [13,14]. More importantly, the accumulated trace metals in river sediments can be released and result in secondary pollution with higher risk [15]. Therefore, both riparian soils and river sediments have become valuable environmental archives for the investigation of anthropogenic contamination. However, less attention has been given to the differences in the characteristics of trace metal pollution, heterogeneity of contents, geochemical fractions, and quantitative contribution of potential pollution sources between riparian soils and river sediments. In general, riparian soils and river sediments have a very close interrelationship. On the one hand, polluted sediments that are transported during high water events onto river terraces contribute significant quantities of trace metals to riparian soils. On the other hand, trace metals absorbed by riparian soils in the flood zone also further infiltrate the soil profile and are drawn into the river sediments [16]. Thus, trace metals are not always deposited downstream of pollution sources; their distribution also depends on fluvial erosion, deposition patterns, and other factors [17,18]. The input source and migration characteristics of trace metals will directly affect their accumulation degree in different environmental media. To further effectively control the formation of metal pollution in the river basin, it is important to clearly understand the pollution characteristics of trace metals in riparian soils and river sediments and to target their potential sources.

Six heavy metals, Pb, Cd, Cu, Cu, Zn, and Ni, in riparian soils and river sediments along the BJR, DJR, and XJR have been systematically analyzed. However, the enrichment characteristics of Pb and Cd were significantly higher than those of other heavy metals after further detailed analysis. Therefore, in order to highlight the key points, this study focuses on the issue of the enrichment status and input sources of Pb and Cd in three lower reaches of the Pearl River Delta located in the GBA. Riparian soils and river sediments in the lower reaches of the BJR, DJR, and XJR were collected systematically to determine their contents, geochemical fractions, and Pb isotopic compositions. The purposes of the research are to (1) evaluate the enrichment status of Pb and Cd in riparian soils and river sediments in three reaches; (2) explore the relationship between enrichment factors of Pb and Cd and their geochemical fraction distribution; and (3) identify the potential input source contributions of Pb and Cd in riparian soils and river sediments in the three reaches.

## 2. Materials and Methods

### 2.1. Study Area and Sample Collection

**Figure 1:**
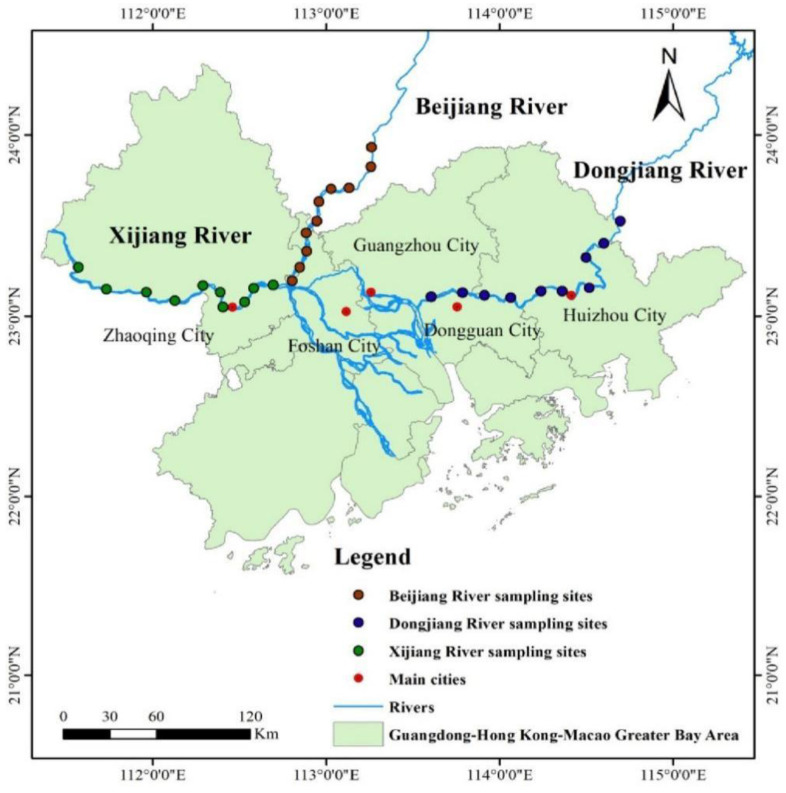
Location of the research site.

The Pearl River has the second largest runoff in China, and its water network is very complex. In general, the Beijiang River (BJR), Dongjiang River (DJR), and Xijiang River (XJR) are the three main tributaries and account for 89.6% of the total flow of the Pearl River [19,20]. Located at the mouth of the lower reaches of the Pearl River, the GBA comprises the two special administrative regions of Hong Kong and Macao and nine cities in the Pearl River Delta (PRD), with a total area of 56,000 square kilometers. By the end of 2018, the total population had reached 70 million, making it one of the most open and economically dynamic regions in China. In this study, 60 samples, including 30 riparian soil samples and 30 river sediment samples, were collected from the lower reaches of the BJR, DJR, and XJR in the GBA (Figure 1). For riparian soil samples, 5 subsamples (0–20 cm in depth) were collected at each sampling location within 20 m of the riverbank and mixed thoroughly to obtain a representative sample. For river sediment samples, surface river sediments (0–20 cm in depth) were collected using a Van Veen stainless steel grab sampler. All collected samples were packed into polyethylene bags and brought back to the laboratory as soon as possible for further analysis. Before determining the experimental scheme, all samples were naturally air-dried at room temperature and then ground to pass through 0.149 mm sieves for total metal concentration and geochemical fraction determination.

### 2.2. Selective Sequential Extraction Experiments

A five-step sequential extraction method was conducted for Pb and Cd geochemical fraction analysis [21]. Specifically, Pb and Cd were divided into five geochemical fractions: the exchangeable fraction (F1), carbonate fraction (F2), iron and manganese oxide fraction (F3), organic matter fraction (F4), and residual fraction (F5). The first step (to obtain F1) involved weighing 2.000 g of the sample into a 50 mL polypropylene centrifuge tube, adding 16 mL of 1 mol/L MgCl_2_ (pH = 7.0) solution, and shaking at room temperature for 1 h. Centrifugal separation (5000 r/min, 10 min) was performed and 1 mL of the supernatant was extracted to test its content. The supernatant was poured out into a polyethylene bottle. Then, 20 mL of deionized water was added to wash the residue, which was shaken for 20 min and centrifuged, and the washing solution was discarded. The second step (to obtain F2) was to add 16 mL of 1 mol/L NaAc (pH = 5.0) solution to the residue of the first step, shake at room temperature for 5 h, and then centrifuge. The rest of the operations were the same as those in the first step. The third step (to obtain F3) was to add 20 mL of 0.04 mol/L NH_2_OH·HCl, shake intermittently at 95 °C for 3 h, and centrifuge. The rest of the operations were the same as those in the first step. In the fourth step (to obtain F4), 3 mL of 0.01 mol/L HNO_3_ and 5 mL of 30% H_2_O_2_ were added to the residue of the third step, and the mixture was heated in a water bath to 85 °C and shaken intermittently for 2 h during this process. Then, 5 mL of H_2_O_2_ was added, and the mixture was heated at 85 °C for 2 h and shaken intermittently; it was cooled to 25 °C, 5 mL NH_4_Ac (2 mol/L, 20% HNO_3_ solution) was added, and the mixture was continuously shaken for 30 min and centrifuged. The rest of the operations were the same as those in the first step. In the fifth step (to obtain F5), 0.100 g of the residue was weighed after the fourth step of extraction and transferred to a 50 mL polytetrafluoroethylene beaker. Then, 10 mL HNO_3_, 1 mL HF, and 1 mL HClO_4_ were added after being covered, and the solution was digested on a hot plate until clear and transparent. The recovery rate of the sequential extraction method was maintained between 80% and 120% to ensure the accuracy of the extraction process. At the same time, parallel samples and duplicate check samples are inserted into the sample to ensure the accuracy and precision of the experiment.

### 2.3. Determination of Pb and Cd Concentrations

To analyze the total metal concentration, 0.1 g of solid sample was pressure-digested in Teflon vessels via a concentrated HF-HNO_3_-HClO_4_ mixture [22]. The concentrations of Pb and Cd were all measured using multicollector–inductively coupled plasma–mass spectrometry (MC–ICP–MS) (Neptune Plus, Thermo Fisher, Waltham, USA). At the same time, for each liquid extract and digested solution from five-step sequential extraction experiments, ICP–MS was used to determine the five fractions of Pb and Cd. The accuracy of Pb and Cd analysis was assessed using reagent blanks, sample duplicates, and standard reference material (GBW07453) from the National Research Center for Standards in China. In addition, the standard deviation of replicated samples was ±5%.

### 2.4. Geoaccumulation Index and Enrichment Factors

The geoaccumulation index (Igeo) was calculated to quantitatively assess the extent of Pb and Cd pollution in the riparian soils and river sediments [23]:

Igeo = log_2_(C_n_/1.5B_n_) (1)

where C_n_ is the measured concentration of Pb and Cd in the riparian soils and river sediments, and B_n_ is the geochemical background value for soil in Guangdong Province [24]. The constant 1.5 was introduced to compensate for possible variations in the background values caused by lithogenic variations [25]. The Igeo values for Pb and Cd were classified into seven levels: uncontaminated (Igeo ≤ 0), uncontaminated to moderately contaminated (0 < Igeo ≤ 1), moderately contaminated (1 < Igeo ≤ 2), moderately to heavily contaminated (2 < Igeo ≤ 3), heavily contaminated (3 < Igeo ≤ 4), heavily to extremely contaminated (4 < Igeo ≤ 5), and extremely contaminated (Igeo > 5).

To further assess the status of Pb and Cd pollution and distinguish between anthropogenic and natural sources, it is useful to calculate the nondimensional enrichment factors (EFs) of Pb and Cd, which are defined by the following equation [26].

EF = (C_i_/C_Fe_)_sample_/(C_i_/C_Fe_)_background_ (2)

where (C_i_/C_Fe_)_sample_ is the ratio of the concentrations of Pb and Cd to that of Fe in samples, and (C_i_/C_Fe_)_background_ is the background ratio of either Pb or Cd to Fe in Guangdong Province.

### 2.5. Pb Isotope Analysis

All samples were crushed to less than 200 mesh and then dissolved in mixed acid (HF-HNO_3_-HClO_4_). After digestion, the samples were stored in 6 M HCl for further separation and purification. Pb was separated and purified by an ion chromatographic column. The ion chromatographic column was filled with 0.5 mL Sr resin and cleaned with 5 mL 3 M HN0_3_, 10 mL HCl, and 5 mL deionized water. Then, 2 mL 3 M HNO_3_ conditioning was applied, followed by Pb separation. The specific steps are as follows: a certain amount of sample was dried and dissolved in a l mL 3 M HNO_3_ upper column, then 3 mL 3 M HNO_3_ was used for elution of matrix elements (K-Na-Ca-Mg), and 2 mL 7 M HNO_3_ and 5 mL 0.05 M HNO_3_ were used for elution of Ba and Sr. Finally, Pb was obtained with 4 mL 8 M HCl and then dried and dissolved in 1 mL 3% HNO_3_ for the next test [27]. Pb isotope ratio measurements were carried out via MC–ICP–MS (Neptune Plus, Waltham, Thermo Fisher, USA) in wet plasma mode at the State Key Laboratory of Environmental Geochemistry, Institute of Geochemistry, Chinese Academy of Sciences. The instrument mass fractionation was corrected by adding internal standard thallium (NIST SRM997, ^205^T1/^203^T1 = 2.3871) and intersample interpolation.

Isotope ratio analysis (IRA) can be applied to identify anthropogenic pollution sources [28]. In this study, the Pb isotope ratios were plotted in a coordinate system using Origin 9.0 software, with different isotopic ratios forming the horizontal and vertical axes. A three-endmember model was used to calculate the relative contribution of natural and two key anthropogenic sources, namely, industrial and agricultural input sources [29]. The equations for the ternary mixing of ^207^Pb/^206^Pb and ^208^Pb/^206^Pb ratios can be written as follows:

^207/206^Pb_s_ = f1 × ^207/206^Pb_f1_ + f2 × ^207/206^Pb_f2_ + f3 × ^207/206^Pb_f3_ (3)

^208/206^Pb_s_ = f1 × ^208/206^Pb_f1_ + f2 × ^208/206^Pb_f2_ + f3 × ^208/206^Pb_f3_ (4)

f1 + f2 + f3 =1(5)

^207/206^Pb_s_ and ^208/206^Pb_s_ are Pb isotope ratios in riparian soil and sediment samples, and ^207/206^Pb_f1_, ^207/206^Pb_f2_ and ^207/206^Pb_f3_ represent Pb isotope ratios from industrial, agricultural, and natural sources, respectively, where f1, f2, and f3 represent the relative contribution rates from industrial, agricultural and natural sources in the sample, respectively.

### 2.6. Multivariate Statistical Analysis

To further clarify the pollution sources of Pb and Cd, multivariate statistical methods were applied in this study. Specifically, Pearson’s correlation analysis (PA) was used to determine how well the sources of trace metals and the route of trace metal pollution were correlated with each other [30]. Then, the PCA method, which is often used to reduce many indicators to a smaller set of comprehensive indicators, was also used in the sample analysis. The validity of PCA was checked using the Kaiser–Meyer–Olkin (KMO) statistic and Bartlett’s test; a KMO value of *p* < 0.5 and a significant Bartlett’s test value were prerequisites [31]. All PA and PCA processes were carried out using SPSS 20 software.

## 3. Results

### 3.1. Distribution of Pb and Cd Contents

The minimum, maximum, and mean values of the total Pb and Cd contents in the riparian soils and river sediments from the BJR, DJR, and XJR are summarized in Table 1. The results showed that mean contents of both Pb and Cd in the BJR riparian soils were significantly higher than those in the XJR and DJR. In particular, the mean contents of Pb and Cd in the BJR sediments were the highest in the three reaches, which is consistent with that in the riparian soils. However, the mean total Cd content in the XJR sediments was significantly higher than that in the DJR sediments and even higher than that in riparian soils along the three reaches.

**Figure 2:**
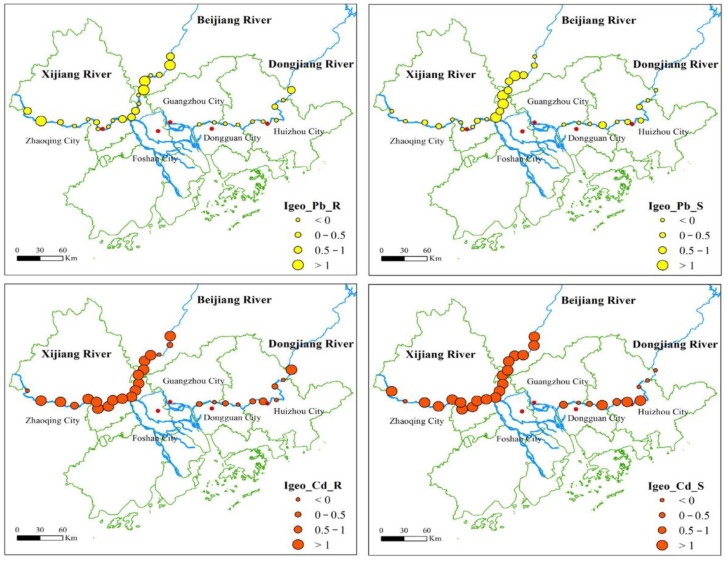
Spatial distribution characteristics of the geoaccumulation index of Pb and Cd in riparian soils and river sediments from three reaches. Note: Igeo_Pb_R and Igeo_Pb_S represent the geoaccumulation index of Pb from riparian soils and river sediments, respectively, and Igeo_Cd_R and Igeo_Cd_S represent the geoaccumulation index of Pb from riparian soils and river sediments, respectively.

The Igeo values of Pb and Cd were calculated in detail. The maximum, minimum, and average values of Pb and Cd in the three rivers are shown in Table S1. Similar to the analysis results of heavy metal content, the accumulation degree of Pb and Cd of BJR was successively higher than that of XJR and DJR. The spatial distribution characteristics of the geoaccumulation index of Pb and Cd in riparian soils and river sediments from the three reaches are shown in Figure 2. The spatial distribution reveals that Pb accumulation in riparian soils mainly occurred in the upper reaches of the BJR and XJR, and Pb accumulation in river sediments mainly occurred in the BJR. For Cd, both the BJR and the XJR are highly enriched overall, and some regions of high accumulation also appear in the middle reaches of the BJR. In particular, from the Igeo values, the average Igeo values of riparian soils were BJR (0.52) > XJR (0.06) > DJR (−0.23) for Pb, while those of Cd were XJR (2.04) > BJR (1.85) > DJR (0.24). In the riparian soils, the average accumulation degree of Pb in the BJR was the highest, while that of Cd in the XJR was the highest.

For river sediments, the average Igeo values followed the order BJR (0.76) > DJR (−0.15) > XJR (−0.28) for Pb, while those of Cd followed the order BJR (3.01) > XJR (1.98) > DJR (0.64). Different from riparian soils, the maximum accumulation of Pb and Cd in river sediments occurred in the BJR. There was little difference between the average accumulation degree of Pb in the XJR and DJR, but the average accumulation degree of Cd in the XJR was significantly higher than that in the DJR.

### 3.2. Distribution of Pb and Cd Geochemical Fractions

**Figure 3:**
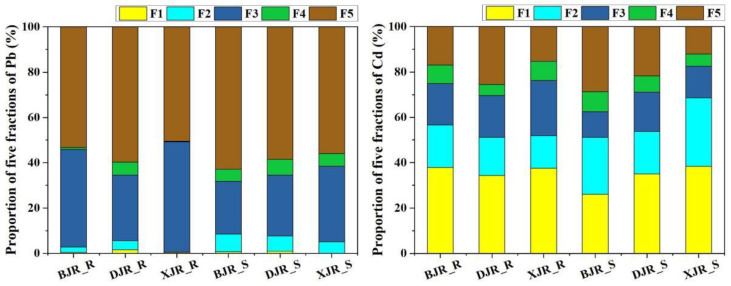
Proportion of five fractions of Pb and Cd in riparian soils and river sediments from three reaches.

The geochemical fractions of Pb and Cd based on a five-step sequential extraction method are shown in Figure 3. The different geochemical fractions of Pb in the riparian soils had a similar distribution, which showed that F1 had the lowest proportion, while F5 had the highest proportion. The mean values of the five geochemical fractions of Pb in riparian soils in the three reaches were F5 (54.5%) > F3 (40.3%) > F4 (2.30%) > F2 (2.2%) > F1 (0.8%). However, Cd and Pb showed great differences in their geochemical fraction distributions; F1 had the largest proportion, and F4 had the smallest proportion. The mean values of the five geochemical fractions of Cd in the riparian soils of the three reaches were F1 (36.6%) > F3 (20.4%) > F5 (19.2%) > F2 (16.6%) > F4 (7.2%).

Compared with riparian soils, the proportion of Pb in river sediments was higher in the carbonate and organic matter fractions, while the proportion of Pb in the iron and manganese oxide fractions was significantly lower than that in riparian soils. Specifically, the mean values of the five geochemical fractions of Pb in river sediments were F5 (59.1%) > F3 (27.9%) > F2 (6.5%) > F2 (5.9%) >F1 (0.5%). For Cd, the carbonate fraction in river sediments was slightly higher than that in riparian soils, while the iron and manganese oxide fraction was slightly lower. The mean values of the five geochemical fractions of Cd in the river sediments of the three reaches were F1 (33.1%) > F2 (24.7%) > F5 (20.9%) > F3 (14.2%) >F4 (7.1%).

### 3.3. Enrichment Factors and Relationship with Nonresidual Fractions

**Figure 4:**
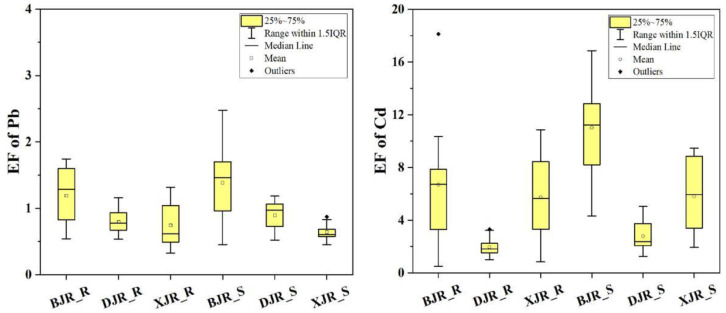
Enrichment factors of Pb and Cd in riparian soils and river sediments from three reaches.

All EF values for Pb and Cd in the three reaches are presented in Figure 4. In riparian soils, the average EF values were in the order BJR (1.19) > DJR (0.79) > XJR (0.74) for Pb, while those of Cd were in the order BJR (6.70) > XJR (5.74) > DJR (1.97). Unlike the results calculated by the Igeo, the results calculated by the enrichment factor showed that Pb and Cd in the riparian soils along the BJR were the highest in the three reaches.

In river sediments, the average EF values followed the order BJR (1.39) > DJR (0.90) > XJR (0.64) for Pb, while those of Cd followed the order BJR (11.02) > XJR (5.83) > DJR (2.79). The order of Pb and Cd enrichment factors in river sediments was consistent with that in riparian soils. The average values of the Pb and Cd enrichment factors in the BJR were the highest in the three reaches, which may indicate that the BJR basin has significantly higher Pb and Cd inputs than the XJR and DJR. To characterize the relationships between Pb–Cd and relevant evaluation indicators (enrichment factors, total contents, and nonresidual fractions of Pb and Cd) in riparian soils and river sediments from the three reaches, the Pearson correlation coefficient matrix of these indicators was calculated, as shown in Table 2.

The results from the correlation coefficients showed that, for riparian soils, the enrichment factor of Pb had a strong positive correlation with the total amount of Pb in the three reaches. However, only the enrichment factor of Pb in the BJR showed a significant positive correlation with the nonresidual fractions of Pb, but not in the DJR and XJR. For Cd in riparian soils, the enrichment factor of Cd had a strong positive correlation with the total amount of Cd in the three reaches. Moreover, the enrichment factors of Cd and Pb were also strongly correlated, which might indicate the similarity of Pb and Cd input sources in riparian soils along the DJR.

For river sediments, there was a strong positive correlation between the Pb enrichment factor and total Pb content in the BJR but not in the DJR and XJR. However, the enrichment factors of Cd in the three reaches were significantly correlated with the total amount of Cd and nonresidual fractions of Cd, and the correlation coefficients of the total amount of Cd and enrichment factors of Cd were higher than those of the nonresidual fractions of Cd and enrichment factors of Cd.

### 3.4. Source Identification by Principal Component Analysis

**Figure 5:**
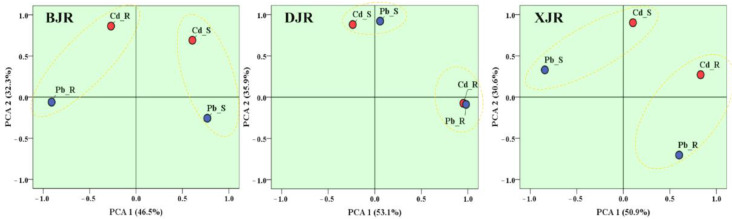
PCA results of Pb and Cd in riparian soils and river sediments from the BJR, DJR, and XJR reaches.

Principal component analysis (PCA) of Pb and Cd was conducted to further explore the input sources of metals, and the results are shown in Figure 5. To directly examine the similarities and differences in the factors controlling the three reaches, two main principal components were extracted. The two main principal components accounted for more than 78.8%, 89%, and 81.5% of the total variance in Pb and Cd in the three reaches. Obviously, the proportion of the two main principal components in the DJR was significantly higher than that in the BJR and XJR, which to some extent indicates that the input sources of Pb and Cd in the DJR may be simpler than those in the other two rivers. This result also indicates that Pb and Cd in the DJR may be controlled by the same source [32].

In general, Pb and Cd in the river sediments of the three reaches were quite different from the source compositions of the riparian soils, which is especially evident in the BJR and XJR. Pb and Cd in the river sediments of the BJR were mainly controlled by principal component 1 (PCA1), while those of riparian soils, especially Cd, were mainly controlled by principal component 2 (PCA2). In the XJR, Pb and Cd in riparian soils were mainly controlled by PCA1, and those of river sediments were mainly controlled by PCA2. It is particularly noteworthy that Cd in riparian soils and river sediments along the BJR are controlled to a large extent and are significantly different from Pb, which may also reflect the difference between the sources of Cd and Pb in BJR. The results of principal component extraction of Pb and Cd from XJR river sediments also indicate that they may be affected by the mixed influence of PCA1 and PCA2.

### 3.5. Source Identification by Pb Isotope Ratios

**Figure 6:**
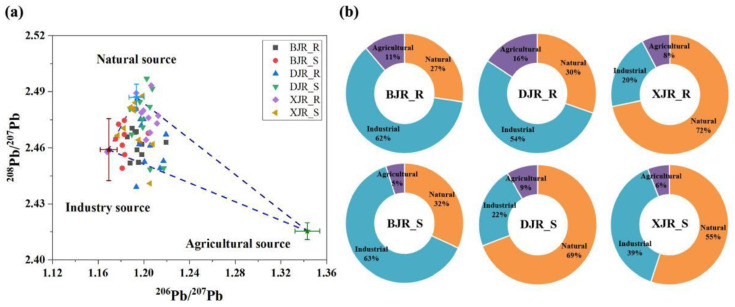
Results of input sources in three reaches based on Pb isotope identification. (**a**) means the ^206^Pb/^207^Pb and ^208^Pb/^207^Pb ratios in the riparian soils and sediments of the three reaches, and (**b**) means the relative contributions of industrial, agricultural, and natural sources in the riparian soils and sediments of the three reaches, respectively.

Industrial, agricultural, and natural sources have different Pb isotopic signatures, and Pb isotope fractionation is little affected by the biochemical reactions occurring during migration; thus, Pb isotope ratios can be used to trace the origin of Pb pollution in riparian soils and river sediments [33,34]. At the same time, Pb isotope fractionation is minimally affected by biochemical reactions during migration. Hence, the ^206^Pb/^207^Pb and ^208^Pb/^207^Pb ratios were used to differentiate between Pb sources in the riparian soils and sediments of the three reaches (Figure 6a). The average ratios of ^206^Pb/^207^Pb in riparian soils from the BJR, DJR, and XJR were 1.195, 1.204, and 1.200, respectively, and those of ^208^Pb/^207^Pb were 2.462, 2.461, and 2.476, respectively. For river sediments, ^206^Pb/^207^Pb and ^208^Pb/^207^Pb were 1.186 and 2.466 in the BJR, 1.201 and 2.475 in the DJR, and 1.193 and 2.472 in the XJR, respectively.

To quantitatively evaluate the contribution of Pb from different sources, a three- endmember mixing model was used in this study. The eigenvalues of Pb isotopes (^206^Pb/^207^Pb and ^208^Pb/^207^Pb) from representative endmembers (industrial endmember, agricultural endmember and natural endmember) appeared to be particularly critical. A comprehensive analysis of the existing Pb isotope studies in the GBA revealed that the average values of ^206^Pb/^207^Pb and ^208^Pb/^207^Pb from industrial sources were 1.169 ± 0.007 and 2.459 ± 0.017, those from agricultural sources were 1.194 ± 0.006 and 2.511 ± 0.018, and those from natural sources were 1.194 ± 0.007 and 2.487 ± 0.007, respectively. Refer to Table S1 for reference values of ^206^Pb/^207^Pb and ^208^Pb/^207^Pb endmember values. Clearly, the ^206^Pb/^207^Pb values of natural sources and agricultural sources are similar, while the ^206^Pb/^207^Pb values of industrial sources appear to be lower than the former two. At the same time, the ^208^Pb/^207^Pb values of the three endmembers were in the following order: agricultural sources > natural sources > industrial sources. Therefore, the strong discrimination of ^206^Pb/^207^Pb and ^208^Pb/^207^Pb from the three endmembers provides support for the quantitative analysis of the contribution of pollution sources [35].

Therefore, there are obvious significant differences in the pollution source structure of Pb in riparian soils and river sediments. A three-endmember model was used to calculate the relative contributions of industrial, agricultural, and natural sources. All the analytical results are shown in Figure 6b. The source identification results showed that in terms of Pb pollution in the riparian soils of the BJR and DJR, industrial pollution was the dominant source (62% and 54%), agricultural pollution was the secondary source (27% and 30%), and the soil parent material contributed the least (11% and 16%). In the XJR, natural sources accounted for 72%, and industrial sources accounted for 20%. For river sediments, the BJR was dominated by industrial sources (63%), while the DJR and XJR were dominated by natural sources (69% and 55%).

## 4. Discussion

### 4.1. Spatial Distribution and Geochemical Fractionation of Pb and Cd

The systematic analysis of the distribution characteristics of the total Pb–Cd contents in riparian soils and river sediments along the BJR, DJR, and XJR revealed that the total contents of Pb and Cd are higher in the three reaches than in most other regions of China. The average Pb and Cd values in the river sediments of the Yangtze River in China are 27.72 mg/kg and 0.30 mg/kg, those of the Yellow River are 19.04 mg/kg and 0.35 mg/kg, and those of the Huaihe River are 29.5 mg/kg and 0.29 mg/kg, respectively [36]. Obviously, the average contents of Pb and Cd in river sediments in the BJR, DJR, and XJR are almost higher than those in these rivers, which indicates excessive accumulation from these metals in the riparian soils and river sediments of the three reaches. Furthermore, the total contents of Pb and Cd in river sediments are significantly higher than those in riparian soils, which all indicates high Pb and Cd contents in the BJR basin. Compared with the soil background values of Pb and Cd in Guangdong Province, the enrichment of Cd and Pb in BJR sediments is 15.8 times and 2.9 times, respectively. The maximum Pb and Cd values also appear in the BJR, and they are 4.6 times and 32.1 times the background values in Guangdong Province, respectively. Unlike riparian soils, river sediments receive trace metals from around the basin, as well as trace metals from secondary pollution from upstream streams [37]. Thus, the metal contents in river sediments are significantly higher than those in riparian soils, which indicates the high enrichment of Pb and Cd in the river sediments along the three reaches. The existence of eigenvalues of ultrahigh contents of Pb–Cd also indicated that there may be corresponding anthropogenic pollution sources that are different from the local soil background [38].

As some of the most persistent pollutants in ecosystems, trace metals may change their geochemical fraction and do not decompose. As a result, the geochemical fractions of metals in riparian soils and sediments are very useful tools for transformation processes and assessing their bioavailability in the studied area is challenging because they can be significantly affected by anthropogenic activities [39]. The systematic analysis of the geochemical fraction distribution characteristics of Pb and Cd in riparian soils and river sediments along the three reaches reveals that Pb is mainly dominated by the residual fraction and that Cd is mainly dominated by the exchangeable fraction, but there are differences in riparian soils and river sediments between different rivers. In general, a high trace metal content in the exchangeable and carbonate fractions leads to more bioavailable metals in both soils and sediments, thereby resulting in more severe toxic effects on living organisms, whereas metals in the residual fraction are not bioavailable and present a low pollution risk [40,41].

Cd is unique, and the dominance of the exchangeable fraction also shows that Cd is very mobile and has the potential to be assimilated by organisms and to impose environmental hazards on ecological systems. A larger proportion of exchangeable Cd in riparian soils and river sediments might be caused by the strong ability to adsorb Cd through electrostatic attraction on the soil or sediment colloid surface [42]. The remarkably high percentages of Cd in the exchangeable and carbonate fractions imply the presence of various anthropogenic sources of Cd in the three reaches. In addition, the proportion of the carbonate fraction in river sediments is significantly higher than that in riparian soils, which can be explained by the fact that Pb and Cd have a special affinity with carbonate and may coprecipitate into carbonate minerals in sediments under an alkaline environment [43].

### 4.2. Heterogeneity of Pb and Cd Enrichment Characteristics in the Three Reaches

The Igeo values indicate that Pb in the riparian soils and river sediments along the BJR is mainly at uncontaminated to moderately contaminated levels, respectively. In contrast, Cd in riparian soils and river sediments is mainly at moderately and heavily contaminated levels, respectively. Pb in riparian soils and river sediments along the DJR is mainly at the uncontaminated level, while Cd is mainly at the uncontaminated to moderately contaminated level. For the XJR, Pb in the riparian soils is mainly at the uncontaminated to moderately contaminated level, while that in the river sediment is mainly at the uncontaminated level; moreover, Cd in the riparian soil is at the moderately to heavily contaminated level, and Cd in the river sediments is mainly at the moderately contaminated level. Obviously, as one of the most hazardous metals to organisms, Cd presents significant enrichment characteristics and pollution in both the riparian soils and sediments along the BJR and XJR. Consequently, the pollution status of the BJR is more severe than that of the other two reaches, and the pollution status of river sediments is more severe than that of riparian soils [44].

Compared with the Igeo, the EF values can better indicate the enrichment behavior of trace metals by eliminating the interference of regional geological background elements by introducing more stable constant reference elements (such as Fe) [45]. In general, the EF values of Pb and Cd in the river sediments of the three reaches are higher than those of the corresponding riparian soils, and the enrichment of Cd is the most obvious. This result is consistent with the results of the previous total content and Igeo analyses. Moreover, the variations in Pb and Cd in riparian soils and river sediments along the BJR are significantly higher than those in the XJR and DJR, which may indicate that outliers increase the overall enrichment degree of the basin. Pb and Cd in riparian soils and river sediments along the DJR are the smallest in the three reaches. At the same time, the average EF value of Cd in riparian soils and river sediments along the XJR is more than 5, which indicates that the high Cd input in the XJR basin cannot be ignored.

To further explore the relationship between the enrichment characteristics of Pb–Cd and their mobility characteristics, Pearson’s correlation analysis (PA) was used to determine the correlation between enrichment factors, total contents, and nonresidual fractions of Pb and Cd in riparian soils and river sediments from the three reaches [46]. The results of the correlation analysis show that the Pb enrichment factor and nonresidual fractions have a significant positive correlation along the BJR, but there is no relationship between the DJR and XJR. However, the enrichment factor of Cd in riparian soils and river sediments along the three reaches shows a significant positive correlation with the nonresidual fractions of Cd. Nonresidual fractions of trace metals are the most easily influenced by human activities, which may further verify that anthropogenic inputs contribute significantly to the enrichment of Cd in the riparian soils and river sediments of the three reaches [47]. A recent study has shown that the additional accumulation of these trace metals (such as Cd, Pb, and Zn) in BJR sediments comes from human activities related mainly to industrial and agricultural effluent from upreach. A similar study was carried out on the Sava River Basin in southeastern Europe and showed that the mean concentrations of trace metals in soils and sediments increased downstream due to increased load from anthropogenic pressures in the lower reach [48]. Therefore, considering that bioavailability and toxicity are dependent on the geochemical fraction and content of trace metals, Cd in riparian soils and river sediments, with the highest EF values, is significantly enriched in the exchangeable fraction and might pose a substantial hazard to the ecology of the three reaches of the GBA [49].

### 4.3. Sources of Cd and Pb in Riparian Soils and River Sediments of the Three Reaches

In river basins, heavy metals are mainly from industrial operations (e.g., mineral mining, tailings leaching, and industrial waste discharge violations), agricultural activities (e.g., chemical fertilizer and pesticide applications), and weathering of soil parent material, all of which can result in heavy metal accumulation in riparian soils and river sediments [50]. Comprehensive correlation analysis and PCA results indicate that the input sources of Pb and Cd in riparian soils and river sediments along the DJR are relatively similar, and the metals are mostly imported from natural sources. However, Pb and Cd sources in riparian soils and river sediments along the BJR and XJR are different, which is dominated by complex mixed sources and reflects the strong influence of human input sources.

China’s modern industry started late, and so did the research on environmental pollution. Research on the heavy metal pollution system of river suspended matter and sediment basically started in the 1990s. At the same time, previous studies have mainly focused on the enrichment status, distribution characteristics, and pollution degree of heavy metal pollution in each river, and analytical research on the source of heavy metal pollution is relatively scarce. In this study, we collected the current status of heavy metal pollution in the rivers of China and other countries in the world. All specific data are shown in Table S2. It is easy to find that the average Pb content in sediments of the Pearl River Basin is significantly higher than that of other rivers, except the Xiangjiang River in China and the Ganga River in India. The average Cd content in sediments is moderate and higher than that of most rivers but lower than that of the Ganga River in India, the Detroit River in America, and the Liaohe River and Xiangjiang River in China. High Pb and Cd contents are often closely related to anthropogenic input. For example, the pollution of Pb and Cd in Xiangjiang River is caused by human mining activities [51].

The BJR basin is composed of different rock types, including granite, limestone, and clay rocks, and runs through the city of Shaoguan, which is well known for its high production of nonferrous metals. Trace metals, such as Cd, Zn, Cu, and Pb, produced by upstream mining can migrate downstream by flowing water and eventually accumulate in river sediments [52]. Different from the main mineral industry in the upper reaches of the BJR, the upper reaches of the DJR are mainly dominated by agriculture and manufacturing. Therefore, Pb and Cd in DJR riparian soils and river sediments mainly come from the joint influence of industry and agriculture. Unlike the BJR and DJR, the upper reaches of the XJR are areas with high geological backgrounds of trace metals, and large amounts of trace metals can be transported downstream through runoff. A new study on a variety of trace metals in terrestrial bedrock, soil and river sediments in the upper reaches of the XJR found that rapid differentiation of bedrock is the main cause of the enrichment in Cd, Cu, and Zn in river sediments in carbonate regions, among which Cd released from black shale is the main source of Cd pollution in the upper reaches of the XJR basin [53].

The multivariate statistical analysis method can qualitatively analyze the possible sources of Pb and Cd in different rivers and soil types and their similarity, but the isotope method must be used to quantitatively analyze the relative contribution rate of each source [54]. In general, the results of PCA showed that Pb and Cd were homologous inputs in the DJR, and there were significant differences only in the riparian soils and river sediments. These results also indicated that Pb and Cd in riparian soils and river sediments along the BJR and XJR were affected by more complex inputs of mixed sources. Furthermore, Pb isotopes can better trace the input sources of trace metal elements under different media conditions in different river basins. All Pb isotopic ratios of ^206^Pb^/207^Pb and ^208^Pb/^207^Pb from industrial, agricultural and natural end members are showed in Table S3. The results of Pb isotope analysis also confirm the results of correlation analysis and principal component analysis. As one of three tributaries of the Pearl River, the BJR, which flows through the most important Fankou Pb–Zn mine and Dabaoshan polymetallic mine in southern China, has been used to transport Pb and Zn to the industries in the PRD [55]. Therefore, industrial sources characterized by mining could be a potential source of anthropogenic Pb in the BJR. On the other hand, according to the impacts of parent material on the distributions of trace metals in Quaternary sediments from the PRD, the alluvial sediments in the PRD had stable sources [56]. Generally, Pb in most southern rivers was predominantly governed by the geological setting, regional mineralization, and geochemical landscape. The main factors determining the high concentrations of Pb in the deep soils of the rivers of the PRD were source rocks and mineral deposits in the river catchment, particle compositions, and the sedimentary environment [57]. These factors may be the main reasons why Pb in DJR and XJR sediments mainly originates from natural sources.

## 5. Conclusions

The contents, geochemical fractions, and input source heterogeneity of Pb and Cd in riparian soils and river sediments from the lower reaches of the BJR, DJR, and XJR showed that all Pb and Cd in riparian soils and river sediments exceeded the background values to varying degrees. The anthropogenic pollution of Pb and Cd was probably from ore-mining activities in the BJR, as indicated by the PCA and Pb isotope compositions. The bedrock with a high geological background from upstream may be the main reason for Cd accumulation in the XJR. The DJR is mainly affected by natural and agricultural sources, and the pollution degree is relatively low. Pb and Cd contamination of the riparian soils and river sediments in BJR and XJR could have significant impacts on the health of the residents and environmental quality of the local terrestrial and aquatic environment, and management strategies should be considered to minimize contamination of rivers.

## Figures and Tables

**Table 1:** Statistical summary of Pb and Cd contents in riparian soils and river sediments from BJR, DJR, and XJR (mg/kg).

Heavy Metal Total Content (mg/kg)		Pb			Cd		
		BJR	DJR	XJR	BJR	DJR	XJR
Riparian soils	Min	45.9	38.4	31.8	0.04	0.1	0.09
	Max	152.7	85.7	126.1	2.08	0.66	1.58
	Mean	95.3	52.8	71.1	0.83	0.21	0.79
	SD	38.03	15.36	29.73	0.64	0.17	0.49
River sediments	Min	48.7	33.8	29.2	0.43	0.12	0.15
	Max	173.3	109.1	85.1	3.21	1.59	1.45
	Mean	107.4	59.2	52.6	1.58	0.38	0.85
	SD	37.33	25.29	16.76	0.75	0.44	0.48

Note: Background value was the environmental background value for the soil of Guangdong Province [24].

**Table 2:** Correlation relationship between enrichment factors, total contents, and nonresidual fractions of Pb and Cd in riparian soils and river sediments from three reaches.

		Pb-EF	Pb-Tc	Pb-Nr	Cd-EF	Cd-Tc	Cd-Nr
Riparian soils	BJR	1	0.882 **	0.805 **	−0.130	−0.119	−0.104
			1	0.907 **	0.182	0.207	0.190
				1	0.185	0.186	0.260
					1	0.993 **	0.950 **
						1	0.957 **
							1
	DJR	1	0.688 *	0.039	0.681 *	0.484	0.390
			1	−0.143	0.728 *	0.887 **	0.885 **
				1	−0.387	−0.375	−0.325
					1	0.872 **	0.742*
						1	0.962 **
							1
	XJR	1	0.957 **	−0.091	−0.009	−0.011	0.100
		0.957 **	1	−0.092	0.230	0.235	0.354
				1	0.262	0.161	0.130
					1	0.990 **	0.977 **
						1	0.984 **
							1
River sediments	BJR	1	0.935 **	0.309	0.127	−0.059	−0.077
			1	0.282	0.283	0.181	0.177
				1	0.074	−0.073	−0.024
					1	0.947 **	0.836 **
						1	0.916 **
							1
	DJR	1	0.613	−0.627	−0.097	−0.044	0.169
			1	−0.162	0.408	0.637*	0.782 **
				1	0.316	0.337	0.168
					1	0.926 **	0.690 *
						1	0.878 **
							1
	XJR	1	0.554	0.166	−0.514	−0.554	−0.437
			1	0.014	−0.071	0.133	0.299
				1	0.375	0.140	0.374
					1	0.937 **	0.818 **
						1	0.894 **
							1

Note: ** indicates a significant correlation at the level of 0.01 (bilateral), * indicates a significant correlation at the level of 0.05 (bilateral); EF, Tc and Nr represent enrichment factors, total contents, and nonresidual fractions of Pb and Cd, respectively.

## Data Availability

The associated dataset of the study is available upon request to the corresponding author.

## Supplementary Materials

The following supporting information can be downloaded at: https://www.mdpi.com/article/10.3390/ijerph192113819/s1, Table S1. Statistical summary of the Igeo values of Pb and Cd in riparian soils and river sediments from BJR, DJR and XJR (mg/kg); Table S2. Pb and Cd contents in sediments of major rivers in the world; Table S3: Pb isotopic ratios of ^206^Pb/^207^Pb and ^208^Pb/^207^Pb from industrial, agricultural and natural end members. References [51,58,59,60,61,62,63,64,65,66,67,68,69,70,71,72,73,74] are cited in the Supplementary Materials.
