# Supplementary material for: Geochemical Fractionation and Source Identification of Pb and Cd in Riparian Soils and River Sediments from Three Lower Reaches Located in the Pearl River Delta
Source: Int J Environ Res Public Health. 2022 Oct 24;19(21):13819. doi: 10.3390/ijerph192113819 (PMC9657673; doi:10.3390/ijerph192113819)
Supplement: Supplementary file 1 [file ijerph-19-13819-s001.zip › ijerph-1950482-supplementary.pdf]

International Journal of
Environmental Research and Public Health

MDPI

Supplementary Materials

Table S1. Statistical summary of the Igeo values of Pb and Cd in riparian soils and river sediments from BJR, DJR and XJR (mg/kg).

Geo-accumulation index (Igeo)		Pb			Cd		
		BJR	DJR	XJR	BJR	DJR	XJR
Riparian soils	Min	-0.29	-0.55	-0.82	-2.1	-0.64	-0.69
	Max	1.44	0.61	1.16	3.79	2.14	3.39
	Mean	0.65	-0.14	0.22	1.82	0.25	2.01
	SD	0.61	0.37	0.61	1.76	0.81	1.26
River sediments	Min	-0.21	-0.78	-0.94	1.51	-0.31	0.04
	Max	1.62	-0.73	0.6	4.42	3.41	3.28
	Mean	0.84	-0.04	-0.16	3.23	0.8	2.18
	SD	0.56	0.59	0.46	0.78	1.15	1.16

Table S2. Pb and Cd contents in sediments of major rivers in the world.

River	Country	Pb (mg/kg)	Cd (mg/kg)
Korotoa [1]	Bangladesh	36-83	0.26-2.8
Ganga [2]	India	148.8-211.4	9.5-79
Sungai [3]	Malaysia	43.4-320	0.2-0.5
Detroit [4]	America	14.5-20.2	1.2-1.6
Ebro [5]	Spain	5.36-33.2	0.10-23.7
Magdalena [6]	Colombia	12.1-18.1	0.11-3.95
Haihe River [7]	China	41.27	0.36
Liaohe River [8]	China	11.54	1.15
Yangtze River [9]	China	39.32	0.4
Xiangjiang River [10]	China	150.3	15.43
Yellow River [11]	China	24.13	0.19
Pearl River (this study)	China	73.07	0.94

Table S3. Pb isotopic ratios of $^{206}Pb/^{207}Pb$ and $^{208}Pb/^{207}Pb$ from industrial, agricultural and natural end members.

Source	Sample description	$^{206/207}Pb$	$^{208/207}Pb$	Reference
Industrial-end member	Pb ore in Guangdong	1.172	2.473	Zhu et al., 2001 [16]
	Dockyard dusts	1.176	2.461	Bi et al., 2013 [12]
	Ironworks dusts	1.178	2.468	Bi et al., 2013 [12]
	Power station dusts	1.173	2.467	Zhu et al., 2001 [16]
	Vehicle exhausted deposits	1.16	2.423	Zhu et al., 2001 [16]
	Hardware factory dusts	1.164	2.46	Zhu et al., 2001 [16]
	Plastic factory dusts	1.161	2.461	Zhu et al., 2001 [16]
Agricultural-end member	Vegetable soils	1.191	2.503	Luo et al., 2012 [14]
	Orchard soils	1.189	2.485	Luo et al., 2012 [14]
	Residual fractions of natural soils	1.203	2.528	Wong et al., 2002 [15]
	Residual fractions of crop soils	1.198	2.517	Wong et al., 2002 [15]
	Residual fractions of paddy soils	1.191	2.524	Wong et al., 2002 [15]
Natural-end member	Granitic rocks	1.184	2.482	Zhu et al., 1998 [17]
	Volcanic rocks	1.199	2.497	Zhu et al., 1989 [18]
	Suburb uncontaminated soils	1.195	2.482	Zhu et al., 2001 [16]
	Alluvial soils	1.196	2.487	Han et al., 2018 [13]

Reference

1. Islam, M.S.; Ahmed, M.K.; Habibullah-Al-Mamun, M.; Hoque, M.F. Preliminary assessment of heavy metal contamination in surface sediments from a river in Bangladesh. *Environ. Earth Sci.* **2015**, *73*, 1837–1848.
2. Gupta, A.; Rai, D.K.; Pandey, R.S.; Sharma, B. Analysis of some heavy metals in the riverine water, sediments and fish from river Ganges at Allahabad. *Environ. Monit. Assess.* **2009**, *157*, 449–458.
3. Nemati, K.; Bakar, N.K.A.; Abas, M.R.; Sobhanzadeh, E. Speciation of heavy metals by modified BCR sequential extraction procedure in different depths of sediments from Sungai Buloh, Selangor, Malaysia. *J. Hazard. Mater.* **2011**, *192*, 402–410.
4. Szalinska, E.; Grgicak-Mannion, A.; Haffner, G.D.; Drouillard, K.G. Assessment of decadal changes in sediment contamination in a large connecting channel (Detroit River, North America). *Chemosphere* **2013**, *93*, 1773–1781.
5. Roig, N.; Sierra, J.; Nadal, M.; Moreno-Garrido, I.; Nieto, E.; Hampel, M.; Gallego, E.P.; Schuhmacher, M.; Blasco, J. Assessment of sediment ecotoxicological status as a complementary tool for the evaluation of surface water quality: The Ebro river basin case study. *Sci. Total Environ.* **2015**, *503*, 269–278.
6. Tejeda-Benitez, L.; Flegal, R.; Odigie, K.; Olivero-Verbel, J. Pollution by metals and toxicity assessment using Caenorhabditis elegans in sediments from the Magdalena River, Colombia. *Environ. Pollut.* **2016**, *212*, 238–250.
7. Zeng, S.; Dong, X.; Chen, J. Toxicity assessment of metals in sediment from the lower reaches of the Haihe River Basin in China. *Int. J. Sediment Res.* **2013**, *28*, 172–181.
8. Ke, X.; Gao, L.; Huang, H.; Kumar, S. Toxicity identification evaluation of sediments in Liaohe River. *Mar. Pollut. Bull.* **2015**, *93*, 259–265.
9. Yi, Y.; Yang, Z.; Zhang, S. Ecological risk assessment of heavy metals in sediment and human health risk assessment of heavy metals in fishes in the middle and lower reaches of the Yangtze River basin. *Environ. Pollut.* **2011**, *159*, 2575–2585.
10. Sun, G.; Wang, X.; Hu, Q. Using stable lead isotopes to trace heavy metal contamination sources in sediments of Xiangjiang and Lishui Rivers in China. *Environ. Pollut.* **2011**, *159*, 3406–3410. https://doi:10.1016/j.envpol.2011.08.037.
11. Ma, X.; Zuo, H.; Tian, M.; Zhang, L.; Meng, J.; Zhou, X.; Min, N.; Chang, X.; Liu, Y. Assessment of heavy metals contamination in sediments from three adjacent regions of the Yellow River using metal chemical fractions and multivariate analysis techniques. *Chemosphere* **2016**, *144*, 264–272.
12. Bi, X.Y.; Liang, S.Y.; Li, X.D. A novel in situ method for sampling urban soil dust: Particle size distribution, trace metal concentrations, and stable lead isotopes. *Environ. Pollut.* **2013**, *177*, 48–57.
13. Han, Z.X.; Wang, X.Q.; Chi, Q.H.; Zhang, B.X.; Liu, D.S.; Wu, H.; Tian, M. Occurrence and source identification of heavy metals in the alluvial soils of Pearl River Delta region, south China. *China Environ. Sci.* **2018**, *38*, 3455–3463.
14. Luo, C.L.; Yang, R.X.; Wang, Y.; Li, J.; Zhang, G.; Li, X.D. Influence of agricultural practice on trace metals in soils and vegetation in the water conservation area along the East River (Dongjiang River), South China. *Sci. Total Environ.* **2012**, *431*, 26–32.

15. Wong, S.C.; Li, X.D.; Zhang, G.; Qi, S.H.; Min, Y.S. Heavy metals in agricultural soils of the Pearl River Delta, South China. *Environ. Pollut.* **2002**, *119*, 33–44.
16. Zhu, B.Q.; Chen, Y.W.; Peng, J.H. Lead isotope geochemistry of the urban environment in the Pearl River Delta. *Appl. Geochem.* **2001**, *16*, 409–417.
17. Zhu, B.Q. *Theory and Application of Isotopic Systematic in Earth Science*; Science Press: Beijing, China, 1998.
18. Zhu, B.Q.; Wang, H.F.; Mao, C.X.; Zhu, N.J.; Huang, R.S.; Peng, J.H.; Pu, Z.P. Geochronology of and Nd-Sr-Pb isotopic evidences for mantle source in the ancient subduction zone beneath Sanshui Basin, Guangdong Province, China. *Chin. J. Geochem.* **1989**, *8*, 65–71.
